# Analysis of Circulating Plasma MicroRNA Profile in Low-Grade and High-Grade Glioma – A Cross-Sectional Study

**DOI:** 10.12688/f1000research.153731.1

**Published:** 2024-11-13

**Authors:** Ery Kus Dwianingsih, Rachmat Andi Hartanto, Sekar Safitri, Yeshua Putra Krisnugraha, Christina Megawimanti Sianipar, Endro Basuki, Kusumo Dananjoyo, Ahmad Asmedi, Bo Sun, Rusdy Ghazali Malueka

**Affiliations:** 1 Department of Anatomical Pathology, Faculty of Medicine, Universitas Gadjah Mada, Dr. Sardjito General Hospital, Special Region of Yogyakarta, 55281, Indonesia; 2 Division of Neurosurgery, Department of Surgery, Faculty of Medicine, Public Health and Nursing, Universitas Gadjah Mada, Dr. Sardjito General Hospital, Special Region of Yogyakarta, 55281, Indonesia; 3 Department of Neurology, Faculty of Medicine, Public Health and Nursing, Universitas Gadjah Mada, Dr. Sardjito General Hospital, Special Region of Yogyakarta, 55281, Indonesia; 4 Nuffield Department of Clinical Neuroscience, University of Oxford, Oxford, England, OX3 7BN, UK

**Keywords:** Glioma; grade; circulating miRNA; blood plasma; NanoString; biomarker

## Abstract

**Background:**

Glioma is the second most common type of brain tumor, accounting for 24% of all brain tumor cases. The current diagnostic procedure is through an invasive tissue sampling to obtain histopathological analysis, however, not all patients are able to undergo a high-risk procedure. Circulating microRNAs (miRNAs) are considered as promising biomarkers for glioma due to their sensitivity, specificity, and non-invasive properties. There is currently no defined miRNA profile that contributes to determining the grade of glioma. This study aims to find the answer for “Is there any significant miRNA that able to distinguish different grades of glioma?”.

**Methods:**

This study was conducted to compare the expression of miRNAs between low-grade glioma (LGG) and high-grade glioma (HGG). Eighteen blood plasma samples from glioma patients and 6 healthy controls were analyzed for 798 human miRNA profiles using NanoString nCounter Human v3 miRNA Expression Assay. The differential expressions of miRNAs were then analyzed to identify the differences in miRNA expression between LGG and HGG.

**Results:**

Analyses showed significant expressions in 12 miRNAs between LGG and HGG, where all of them were downregulated. Out of these significant miRNAs, miR-518b, miR-1271-3p, and miR-598-3p showed the highest potential for distinguishing HGG from LGG, with area under curve (AUC) values of 0.912, 0.889, and 0.991, respectively.

**Conclusion:**

miR-518b, miR-1271-3p, and miR-598-3p demonstrate significant potentials in distinguishing LGG and HGG.

## Introduction

Glioma is the second most common type of brain tumor, accounting for 24% of all brain tumor cases, with meningioma being the most common. ^1^ Glioma is a broad term that encompasses many different subtypes, but it is typically divided into two main categories: low-grade glioma (LGG) and high-grade glioma (HGG), which are classified by the World Health Organization (WHO) as Grade 1-2 and Grade 3-4, respectively. Glioblastoma (GBM) is one of the most aggressive and malignant forms of high-grade glioma. Despite significant advances in diagnostic and treatment technologies, the mortality rate for glioblastoma remains high, with an average life expectancy of only 1.5 years. ^2^ Currently, most cases of glioma are detected using magnetic resonance imaging (MRI), followed by surgery to obtain a tissue sample for histopathological analysis to determine the subtype and grade of the tumor. However, there is a significant problem with inter-observer variability in both radiological and histopathological analysis, which can lead to diagnostic errors. ^3^ In addition, sampling errors can occur if the tumor is not biopsied adequately. ^4^ This invasive diagnostic procedure poses many risks to patients, and not all patients are able to undergo it.

MicroRNA (miRNA) is a small endogenous mediator for RNA interference and a crucial regulatory component for biological development and metabolism. ^5, 6^ It is a group of 18-25 nucleotides that exhibits distinct expression patterns in various types of cancer tissue. ^7, 8^ Many miRNAs demonstrate tissue-specific expression patterns and can exhibit either oncogenic or suppressive properties depending on the processes. ^9^ Moreover, miRNA can cross the blood-brain barrier and exist stably in peripheral circulation. Therefore, miRNA is a strong candidate for a circulatory biomarker for brain tumors, specifically glioma. ^10^ Multiple studies have demonstrated the use of single miRNA or a combination of multiple miRNAs for diagnostic and prognostic purposes in GBM. ^11^ For instance, a study by Yang et al. identified significant differences in seven miRNAs between high-grade astrocytoma and normal control. ^12^ Another study by Roth et al. showed the upregulation of miR-128 and downregulation of miR-342-3p in GBM patients. ^13^

Plasma miRNA is a promising diagnostic tool that is less invasive and has more specific targets. The current WHO approach to glioma diagnosis extensively involves molecular profiling. However, there is inadequate evidence for miRNA profiling in Indonesia, which highlights the need to increase knowledge and awareness of miRNA profiling as a promising modality for glioma diagnosis. This study aims to analyze the existing miRNAs and compare them between LGG and HGG and find the answer for “Is there any significant miRNA that able to distinguish different grades of glioma?”. The results may determine any significant miRNA that are uniquely expressed in either HGG or LGG, which can help in accurate diagnosis of glioma.

## Methods

### Ethical statement

Written informed consent was obtained from the patients or from the patients’ families if the patients were deemed incapable of giving consent (decreased consciousness, severe cognitive impairment). The entire study was approved on 26 May 2023 and supervised by the Medical and Health Research Ethics Committee (MHREC), Universitas Gadjah Mada (UGM), Indonesia (Ref: KE/FK/0878/EC/2023). All ethical considerations were conducted and supervised with a strict relevant guideline and regulation.

### Research subjects

This study retrospectively collected data on newly-diagnosed cases of glioma (n = 18) from Dr. Sardjito General Hospital and its satellite hospitals in Yogyakarta, Indonesia. The data was collected over a period of three years (July 2019-July 2022). Additionally, control samples of healthy people (n = 6) were acquired during this data collection. The cases were divided into two groups based on the 2016 WHO Classification of CNS Tumors, which is LGG and HGG. The diagnosis of glioma, including its histopathology, was confirmed by expert neuropathologists from the Department of Anatomical Pathology at Dr. Sardjito General Hospital. Each patient received personalized standardized therapy based on their diagnosis and clinical condition. Basic demographic, clinical, and supportive examinations, such as pathology and radiology, were obtained from medical records. Preoperative blood samples were taken during tumor resection procedures. The entire study protocol was approved by the Medical and Health Research Ethics Committee (MHREC), Universitas Gadjah Mada (UGM), Indonesia (Ref: KE/FK/0878/EC/2023).

### Obtaining peripheral blood sample

Blood samples were taken from a peripheral vein or artery using ethylenediamine-tetra acetic acid (EDTA) anticoagulated tubes. To ensure anonymity, all samples were coded according to the ethics protocol. The sample was immediately centrifuged at 3000×g for 10 minutes, and the plasma was separated and stored at -80°C for further analysis. Additionally, the Buffy coat was also extracted and kept for future study purposes.

### RNa isolation and purification for nanostring assay

The plasma samples were thawed at room temperature. Afterwards, total cell-free RNA was extracted from plasma and purified by using miRNeasy Serum/Plasma Advanced Kit (Cat. no. 217204) (Qiagen, Germany). For every 200 μl of plasma sample, 60 μl of buffer RPL containing guanidine thiocyanate and detergents was mixed thoroughly and kept in room temperature for 2-3 minutes before adding 20 μl of buffer RPP. Another vigorous mixing and 3-minute room temperature incubation (15-25°C) were performed. The sample was centrifuged at 12000 g for 3 minutes and should produce a clear and colorless supernatant. 1 volume of isopropanol was added to the supernatant and centrifuged at ≥8000 g for 15 seconds followed by addition of 700 μl of buffer RWT and centrifugation at ≥8000 g for 15 seconds. Next, 500 μl of buffer RPE was mixed and centrifuged again at ≥8000 g for another 15 seconds. Lastly, the mixture was mixed with 50 μl of 80% ethanol before centrifugation at ≥8000 g for 2 minutes. The sample was then washed with 20 μl of RNAse-free water and incubated for 1 minute before 1-minute full speed centrifugation to completely elute the RNA. The final mixture was ready to be used for further analysis. Each step was performed according to the standard protocol issued by the manufacturer. To assess the quality of the RNA, a Nanodrop device (Thermo Scientific, Waltham, MA, USA) was used.

### Nanostring nCounter Assay and Data Analysis

A NanoString nCounter Human v3 miRNA Expression Assay (Cat. no. CSO-MIR3-12) (NanoString Technologies, Seattle, WA, USA) was conducted on all samples. The assay used 798 unique miRNA barcodes. To perform the analysis, 100 ng total cell-free RNA from each sample was mixed with pairs of capture and reporter probes customized for specific recognition of each miRNA presence. Overnight hybridization at a temperature of 65°C allowed sequence-specific probes to form complexes with targets. Two-step magnetic-beads-based purification on an automated fluidic handling system (nCounter Prep Station, Thermo Scientific, Waltham, MA, USA) was used to remove excess probes, and target-probe complexes were immobilized on the cartridge for data collection. Data collection was carried out on the nCounter Digital Analyzer (NanoString Technologies, Seattle, WA, USA) following the manufacturer’s instructions, to count individual fluorescent barcodes and quantify target RNA molecules present in each sample. For each assay, a high-density scan (600 fields of view) was performed.

The NanoString raw data or Reporter Code Counts (RCC) file was analyzed using nCounter analysis software developed by ROSALIND, Inc (ROSALIND, San Diego, CA) ( https://rosalind.bio/) which employs a HyperScale architecture for bioinformatics problem solving. As part of the quality control step, ROSALIND generated Read Distribution percentages, violin plots, identity heatmaps, and sample MDS plots. Nanostring’s criteria were used to calculate normalization, fold changes, and *p-value *s. Background subtraction was performed based on POS_A probe correction factors, followed by normalization in two steps: positive control normalization and codeset normalization. During both normalization steps, the geometric mean of each probeset was used to create a normalization factor. ROSALIND employed the t-test method to calculate fold changes and p-values for comparisons. *p-value* adjustment was performed using the Benjamini-Hochberg method to estimate false discovery rates (FDR). For the final heatmap of differentially expressed miRNA, clustering was done using the PAM (Partitioning Around Medoids) method with the fpc R library. This method takes into consideration the direction and type of all signals on a pathway, as well as the position, role, and type of every miRNA. As an alternative software, an open-source bioinformatics tool is provided by Illumina in GitHub and is available to use ( https://github.com/Illumina).

### Statistical analysis

Relevant data were extracted from ROSALIND and statistical analysis was performed using IBM SPSS Ver. 26 using glioma grading and miRNA reposited dataset ^14^ to generate demographic data and analysed the correlation between LGG and HGG with candidate miRNA. The first step was to generate boxplots for all unique significantly expressed miRNAs based on ROSALIND analysis. *p-value* was determined using Kruskal Wallis Test. ROC-AUC graph were then generated using the normalized expression of candidate miRNA. All significant miRNAs were subjected to univariate analysis using Mann-Whitney Test to determine their correlation with glioma grading. A *p-value* of less than 0.05 was considered significant in all tests.

## Results

In this study, a total of 24 subjects were enrolled. Out of these, 6 individuals (25%) were healthy controls, while 6 (25%) were diagnosed with diffuse astrocytoma, 6 (25%) with anaplastic astrocytoma, and another 6 (25%) with GBM ( Table 1). This means that among the tumor group, 6 (33.3%) were classified as LGG, and 12 (66.6%) as HGG. Among the subjects with tumors, 11 (61.1%) were male and 7 (38.9%) were female. Most of them (15 samples) were under the age of 60, while the remaining 3 (16.7%) were over 60. The tumors were mostly located in the frontal lobe, while some involved parietal and/or temporal lobes. Twelve (66.7%) of the tumors had a wildtype IDH1, while the other 6 (33.3%) had a mutant IDH1.

**Table 1.** Baseline characteristics of the tumor group (n = 18).

Sample ID	Age	Gender	Diagnosis	Grade
**Low Grade Glioma**				
FG – 203	26	M	Diffuse Astrocytoma	II
FG – 197	36	F	Diffuse Astrocytoma	II
FG – 219	40	M	Diffuse Astrocytoma	II
FG – 161	48	M	Diffuse Astrocytoma	II
FG – 188	47	M	Diffuse Astrocytoma	II
FG – 204	27	M	Diffuse Astrocytoma	II
**High Grade Glioma**				
FG – 206	64	M	Anaplastic Astrocytoma	III
FG – 231	33	M	Anaplastic Astrocytoma	III
FG – 136	38	M	Anaplastic Astrocytoma	III
FG – 242	33	F	Anaplastic Astrocytoma	III
FG – 193	58	F	Anaplastic Astrocytoma	III
FG – 151	69	M	Anaplastic Astrocytoma	III
FG – 163	59	M	Glioblastoma	IV
FG – 190	15	F	Glioblastoma	IV
FG – 172	64	F	Glioblastoma	IV
FG – 224	40	F	Glioblastoma	IV
FG – 158	51	M	Glioblastoma	IV
FG – 233	43	F	Glioblastoma	IV

A total of 21 miRNAs were significantly observed in the samples, out of which 5 were upregulated, and 16 were downregulated ( Table 2). MiR-223-3p exhibited the highest fold changes as compared to other upregulated miRNAs. However, the adjusted *p-value* did not show significance ( *p*=0.088). On the other hand, miR-1271-3p exhibited the biggest fold change with a value of -2.249 compared to other downregulated miRNAs, with an adjusted *p-value* of 0.010.

**Table 2.** Upregulated and downregulated miRNAs.

miRNA	Fold change	*p-value * *	Adjusted *p-value * *
**miR-25-3p**	3.358	0.00198	0.058
**miR-93-5p**	1.783	0.00294	0.069
**miR-106a-5p+hsa-miR-17-5p**	2.061	0.0048	0.077
**miR-15a-5p**	1.804	0.0134	0.086
**miR-223-3p**	4.844	0.0142	0.088
**miR-107**	-1.993	0.00000113	0.001
**miR-518b**	-2.206	0.000035	0.010
**miR-1271-3p**	-2.249	0.0000513	0.010
**miR-598-3p**	-2.169	0.0000517	0.010
**miR-216a-5p**	-2.110	0.0000897	0.011
**miR-6721-5p**	-2.228	0.0000919	0.011
**miR-23c**	-2.047	0.000108	0.011
**miR-4443**	-1.886	0.000114	0.011
**miR-873-3p**	-2.055	0.000206	0.018
**miR-363-3p**	-1.951	0.000307	0.024
**miR-421**	-1.539	0.000591	0.040
**miR-127-5p**	-1.963	0.000604	0.040
**miR-495-3p**	-1.703	0.000711	0.044
**miR-887-5p**	-1.747	0.00087	0.046
**miR-1286**	-1.744	0.000885	0.046
**miR-550a-5p**	-2.007	0.000924	0.46

* *p-value* calculated by ROSALIND ^®^, <0.05 is considered statistically significant.

**Figure 1.**
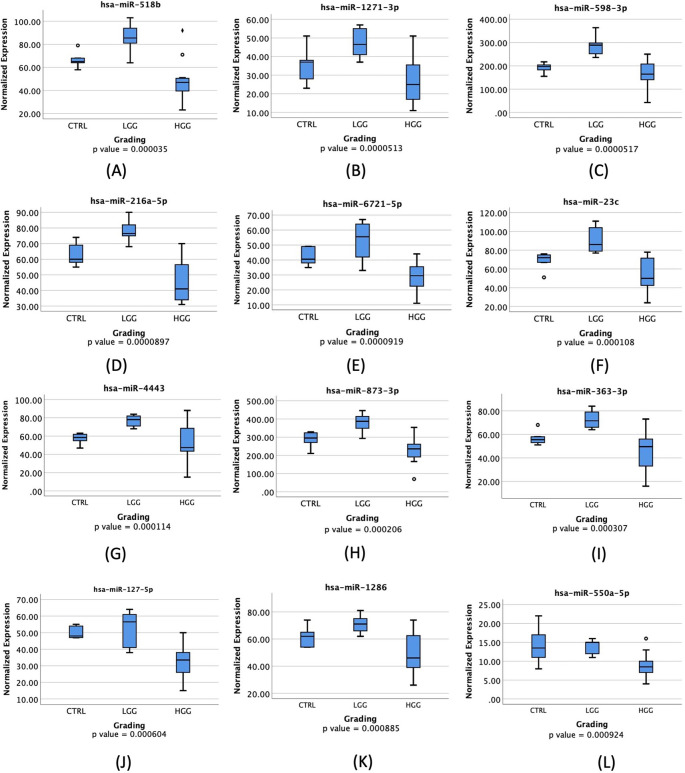
Boxplot of miRNAs normalized expression; Analysis using Kruskal Wallis Test, *p-value* <0.05 is considered significant. ♦: extreme outlier; ο: mild outlier; CTRL: control; LGG: low-grade glioma; HGG: high-grade glioma.

These significantly expressed miRNAs were further analyzed to determine their ability to distinguish between HGG and LGG. These 12 miRNAs will be further represented as box plots in Figures 1A- 1L to provide a visual representation of their significance.

**Figure 2.**
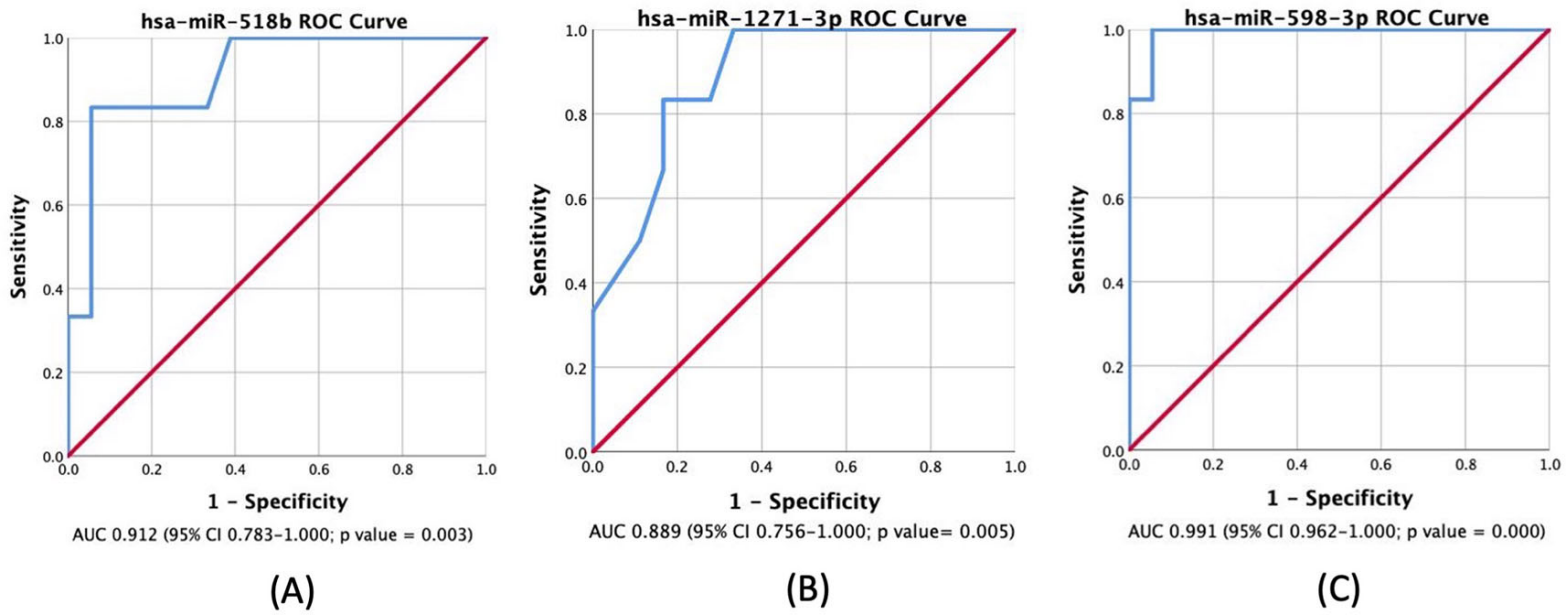
ROC curve and analysis of upregulated and downregulated miRNAs. The positive actual state is high-grade glioma (HGG); *p-value* <0.05 is considered statistically significant. AUC: Area Under Curve; CI: Confidence Interval.

Most miRNAs were highly expressed in LGG compared to HGG and control groups, with all showing a significant *p-value* of less than 0.05 as depicted in Figure 1. Table 3 displays the AUC results for the miRNAs analyzed. According to the results, miR-518b, miR-1271-3p, and miR-598-3p showed AUC values of 0.912, 0.889, and 0.991, respectively. These miRNAs have a high true positive rate for distinguishing between LGG and HGG. The ROC Curve will be individually displayed for miR-518b ( Figure 2A), miR-1271-3p ( Figure 2B), and miR-598-3p ( Figure 2C). On the other hand, the AUC values for miR-216a-5p, miR-6721-5p, miR-23c, miR-4443, miR-363-3p, miR-873-3p, miR-127-5p, miR-1286, and miR-550a-5p were low.

**Table 3.** ROC curve analysis of upregulated and downregulated miRNAs.

No	miRNA profile	AUC * (95% CI)	*p-value of AUC*	*p-value * of univariate analysis **
1	hsa-miR-518b	0.912 (0.783-1.000)	0.003 ^α^	0.019 ^α^
2	hsa-miR-1271-3p	0.889 (0.756-1.000)	0.005 ^α^	0.036 ^α^
3	hsa-miR-598-3p	0.991 (0.962-1.000)	0.000 ^α^	0.153
4	hsa-miR-216a-5p	0.097 (0.000-0.219)	0.001 ^α^	0.155
5	hsa-miR-6721-5p	0.076 (0.000-0.181)	0.000 ^α^	0.051
6	hsa-miR-23c	0.149 (0.000-0.302)	0.004 ^α^	0.260
7	hsa-miR-4443	0.247 (0.037-0.456)	0.035 ^α^	0.048 ^α^
8	hsa-miR-363-3p	0.167 (0.000-0.335)	0.006 ^α^	0.051
9	hsa-miR-873-3p	0.146 (0.000-0.308)	0.003 ^α^	0.032 ^α^
10	hsa-miR-127-5p	0.063 (0.000-0.158)	0.000 ^α^	0.026 ^α^
11	hsa-miR-1286	0.198 (0.009-0.386)	0.012 ^α^	0.046 ^α^
12	hsa-miR-550a-5p	0.135 (0.000-0.292)	0.002 ^α^	0.023 ^α^

The positive actual state is high-grade glioma (HGG); *p-value* <0.05 is considered statistically significant.

* AUC: Area Under Curve.

^α^ *p-value* <0.05.

** Mann-Whitney Test.

Univariate analysis was conducted using the Mann-Whitney test to compare the expression of miRNAs between HGG and LGG. The results indicated that miR-518b, miR-1271-3p, miR-4443, miR-873-3p, miR-127-5p, miR-1286, and miR-550a-5p have significantly lower expression in HGG compared to LGG (with a *p-value* of less than 0.05).

## Discussion

This study has identified several circulating plasma miRNAs that exhibit significantly lower expression in HGG as compared to LGG. Notably, miR-518b, miR-1271-3p, and miR-598-3p demonstrated high AUC values. There is still limited data available on circulating miRNA profiling in glioma. Dysregulation of miRNA has been observed to contribute to various aspects of gliomagenesis, including regulation of cancer stem cells, cell cycle control, apoptosis, angiogenesis, and immune modulation. ^15^

MiR-518b is a type of miRNA that has been found to be significantly expressed in patients with hepatocellular carcinoma (HCC). It has also been associated with the prognosis of HCC patients. However, there is no study linking this miRNA with glioma patients. ^16^ A box plot, shown in Figure 1A, displays a noticeable increase in its expression in LGG patients when compared to HGG patients. Additionally, it shows a 91.2% AUC in ROC Curve, as visualized in Figure 2A, which means that this miRNA has a high capability in distinguishing between LGG and HGG based on its expression level. A previous study found that miR-518a-3p downregulation has a role in decreasing NF-kB inducing kinase (NIK) protein levels in patients with colorectal cancer. This, in turn, lowers NF-kB activity and increases resistance to apoptosis. ^17^ In glioma patients, NF-kB is activated more in HGG. ^18^ NF-kB has a role in mediating inflammatory cytokines regulation, and a study has found an increased miR-518 expression in human endothelial cells that have been exposed to oxidative stress and inflammation. These findings were done in patients with ischemic cerebral infarction. ^19^ Therefore, a higher expression of miR-518b in LGG may associate with a higher NF-kB suppression through NIK downregulation, resulting in a lower grade of glioma.

MiR-1271-3p has been studied and found to have a direct pathway in regulating calcium/calmodulin-dependent protein kinase 2 (CaMKK2). ^20^ It also contributes, together with zinc finger antisense 1 (ZFAS1) and hexokinase 2 (HK2), in regulating growth in glioma tissues. ^21^ Both studies showed that lower expression of CaMKK2 and ZFAS1 were associated with upregulation in miR-1271-3p while interacting with HK2, therefore constraining proliferation, migration, and apoptosis of tumor cells. This is consistent with a significantly higher miR-1271-3p expression in LGG compared to HGG, including GBM, as shown in Figure 1B. Several studies involving various cancers also showed a significant association between miR-1271 overexpression and cell growth inhibition, as seen in oral squamous cell carcinoma (OSCC) and ovarian cancer. ^22, 23^ Both studies implicate that lower expression of miR-1271 will promote cancer cell growth and even metastatic possibility.

There have been limited studies on the activity of hsa-miR-598-3p in tumor regulation. However, one study found a connection between miR-598 and the Metastasis Associated Colon Cancer (MACC1) gene through direct inhibition. MACC1 is highly expressed in GBM, and miR-598 was found to have a direct impact on inhibition of proliferation and invasion through MACC1. ^24^ This suggests that in HGG patients, including GBM, lower levels of miR-598 will be detected compared to LGG due to its natural inhibitive nature. This study supports previous findings as seen in Figure 1C. While not many studies have focused on miR-598 and glioma patients, numerous other studies have revealed that miR-598 has a natural property of inhibiting cell growth and proliferation. Downregulated miR-598 has also been linked to inhibition of gastric cancer cells proliferation, migration, and apoptosis in cases of gastric cancer (GC), osteosarcoma, non-small cell lung carcinoma (NSCLC), and colorectal cancer metastasis. ^25– 27^ Consequently, cancer patients with lower expression of miR-598 will most likely have higher severity or grading compared to low-grade cancer patients, including glioma. Despite significant findings, this study is limited to a small sample size, therefore, another study with larger sample size and more controlled variables needs to be conducted in order to achieve a more conclusive result.

## Conclusion

In conclusion, this study has demonstrated that miR-518b, miR-1271-3p, and miR-598-3p are plasma miRNAs that possess the ability to differentiate between low-grade and high-grade glioma. These miRNAs are overexpressed in low grade gliomas, which is consistent with their role as tumor suppressors. However, this study’s limitation lies in its small sample size, which highlights the need for further validation in a larger group of glioma patients.

## Declarations

### Ethical statement

Written informed consent was obtained from the patients or from the patients’ families if the patients were deemed incapable of giving consent (decreased consciousness, severe cognitive impairment). The entire study was approved on 26 May 2023 and supervised by the Medical and Health Research Ethics Committee (MHREC), Universitas Gadjah Mada (UGM), Indonesia (Ref: KE/FK/0878/EC/2023).

### Patients consent for publication

Written informed consent was obtained from the patients or from the patients’ families if the patients were deemed incapable of giving consent (decreased consciousness, severe cognitive impairment), including the liberty to use patients’ demographic and clinical information for publication without disclosing patients’ identities. Consent to publish was communicated to the patients or the patients’ families together with consent to participate in the study.

### Preregistered data analysis

This study did not preregister any data analysis plan at an independent registry.

## Software availability

ROSALIND and IBM SPSS Ver. 26 were used for nCounter analysis and statistical analysis, respectively. ROSALIND is a subscription-based tool developed by ROSALIND, Inc ( https://rosalind.bio/). Alternatively, GitHub by Illumina provides various open-source bioinformatics tools which is available for use ( https://github.com/illumina).

## Data Availability

Harvard Dataverse: Integrated Datalog MicroRNA and Glioma Grade https://doi.org/10.7910/DVN/N3WTWV. ^28^ The dataset contains following underlying data:
• Full Data Nanostring (290423).tab (Include subject ID, baseline characteristics, glioma subtype and location, and normalized MiRNA expressions) • MiRNA Glioma Grade 2 Only.tab (control sample vs glioma subjects grade 2 normalized MiRNA expressions) • MiRNA Glioma Grade 3 Only.tab (control sample vs glioma subjects grade 3 normalized MiRNA expressions) • MiRNA Glioma Grade 4 Only.tab (control sample vs glioma subjects grade 4 normalized MiRNA expressions) Data are available under the terms of Creative Commons CC0 1.0 Universal Public Domain Dedication.

## References

[1] Ostrom QT Gittleman H Truitt G : CBTRUS statistical report: Primary Brain and other central nervous system tumors diagnosed in the United States in 2011–2015. *Neuro-Oncology.* 2018;20(suppl_4):iv1–iv86. 10.1093/neuonc/noy131 30445539 PMC6129949

[2] Van Meir EG Hadjipanayis CG Norden AD : Exciting new advances in neuro-oncology: The avenue to a cure for malignant glioma. *CA Cancer J. Clin.* 2010;60(3):166–193. 10.3322/caac.20069 20445000 PMC2888474

[3] Beyer S Fleming J Meng W : The role of mirnas in angiogenesis, invasion and metabolism and their therapeutic implications in gliomas. *Cancers.* 2017;9(7):85. 10.3390/cancers9070085 28698530 PMC5532621

[4] Drusco A Bottoni A Laganà A : A differentially expressed set of micrornas in cerebro-spinal fluid (CSF) can diagnose CNS malignancies. *Oncotarget.* 2015;6(25):20829–20839. 10.18632/oncotarget.4096 26246487 PMC4673232

[5] Tomaru Y Hayashizaki Y : Cancer research with non-coding RNA. *Cancer Sci.* 2006;97(12):1285–1290. 10.1111/j.1349-7006.2006.00337.x 17052264 PMC11158021

[6] Chan JA Krichevsky AM Kosik KS : Data from MicroRNA-21 is an antiapoptotic factor in human glioblastoma cells. 2023. 10.1158/0008-5472.c.6493641.v1 16024602

[7] Gourlay J Morokoff AP Luwor RB : The emergent role of exosomes in glioma. *J. Clin. Neurosci.* 2017;35:13–23. 10.1016/j.jocn.2016.09.021 27771233

[8] Mitchell PS Parkin RK Kroh EM : Circulating micrornas as stable blood-based markers for cancer detection. *Proc. Natl. Acad. Sci.* 2008;105(30):10513–10518. 10.1073/pnas.0804549105 18663219 PMC2492472

[9] Svoronos AA Engelman DM Slack FJ : Oncomir or tumor suppressor? the duplicity of micrornas in cancer. *Cancer Res.* 2016;76(13):3666–3670. 10.1158/0008-5472.can-16-0359 27325641 PMC4930690

[10] Morokoff A Jones J Nguyen H : Serum microrna is a biomarker for post-operative monitoring in Glioma. *J. Neuro-Oncol.* 2020;149(3):391–400. 10.1007/s11060-020-03566-w 32915353

[11] Géczi D Nagy B Szilágyi M : Analysis of circulating MIRNA profile in plasma samples of glioblastoma patients. *Int. J. Mol. Sci.* 2021;22(10):5058. 10.3390/ijms22105058 34064637 PMC8151942

[12] Yang C Wang C Chen X : Identification of seven serum micrornas from a genome-wide serum microrna expression profile as potential noninvasive biomarkers for malignant astrocytomas. *Int. J. Cancer.* 2012;132(1):116–127. 10.1002/ijc.27657 22674182

[13] Roth P Wischhusen J Happold C : A specific MIRNA signature in the peripheral blood of glioblastoma patients. *J. Neurochem.* 2011;118(3):449–457. 10.1111/j.1471-4159.2011.07307.x 21561454

[14] Malueka RG : Integrated Datalog MicroRNA and Glioma Grade.[Dataset]. *Harvard Dataverse.* 2024;V1. 10.7910/DVN/N3WTWV

[15] Zhao H Shen J Hodges TR : Serum microrna profiling in patients with glioblastoma: A survival analysis. *Mol. Cancer.* 2017;16(1):59. 10.1186/s12943-017-0628-5 28284220 PMC5346242

[16] Yerukala Sathipati S Ho S-Y : Novel mirna signature for predicting the stage of hepatocellular carcinoma. *Sci. Rep.* 2020;10(1):14452. 10.1038/s41598-020-71324-z 32879391 PMC7467934

[17] Maubach G Feige MH Lim MCC : NF-Kappab-inducing kinase in cancer. Biochimica et Biophysica Acta (BBA) - Reviews on. *Cancer.* 2019;1871(1):40–49. 10.1016/j.bbcan.2018.10.002 30419317

[18] Puliyappadamba VT Hatanpaa KJ Chakraborty S : The role of NF-κB in the pathogenesis of Glioma. *Mol. Cell. Oncol.* 2014;1(3):e963478. 10.4161/23723548.2014.963478 27308348 PMC4905061

[19] Zhao B Jiang X : HSA-mir-518-5p/HSA-mir-3135b regulates the REL/sod2 pathway in ischemic cerebral infarction. *Front. Neurol.* 2022;13. 10.3389/fneur.2022.852013 35481271 PMC9038098

[20] Huang Y-K Su Y-F Lieu A-S : Mir-1271 regulates glioblastoma cell proliferation and invasion by directly targeting the CAMKK2 gene. *Neurosci. Lett.* 2020;737:135289. 10.1016/j.neulet.2020.135289 32791096

[21] Zhang B Chen J Cui M : LncRNA ZFAS1/mir-1271-5p/HK2 promotes glioma development through regulating proliferation, migration, invasion and apoptosis. *Neurochem. Res.* 2020;45(12):2828–2839. 10.1007/s11064-020-03131-x 32964288

[22] Huang S Huang P Wu H : Linc02381 aggravates breast cancer through the mir-1271-5p/FN1 axis to activate PI3K/akt pathway. *Mol. Carcinog.* 2021;61(3):346–358. 10.1002/mc.23375 34882856

[23] Liu X Ma L Rao Q : Mir-1271 inhibits ovarian cancer growth by targeting cyclin G1. *Med. Sci. Monit.* 2015;21:3152–3158. 10.12659/msm.895562 26477861 PMC4617187

[24] Wang N Zhang Y Liang H : MicroRNA-598 inhibits cell proliferation and invasion of glioblastoma by directly targeting metastasis associated in colon cancer-1 (MACC1). *Oncol. Res.* 2018;26(8):1275–1283. 10.3727/096504018x15185735627746 29444745 PMC7844726

[25] Liu N Yang H Wang H : Mir-598 acts as a tumor suppressor in human gastric cancer by targeting IGF-1R. *Onco. Targets. Ther.* 2018;11:2911–2923. 10.2147/ott.s166597 29844688 PMC5961641

[26] Yang F Wei K Qin Z : Mir-598 suppresses invasion and migration by negative regulation of derlin-1 and epithelial-mesenchymal transition in non-small cell lung cancer. *Cell. Physiol. Biochem.* 2018;47(1):245–256. 10.1159/000489803 29768262

[27] Chen J Zhang H Chen Y : Mir-598 inhibits metastasis in colorectal cancer by suppressing Jag1/NOTCH2 pathway stimulating EMT. *Exp. Cell Res.* 2017;352(1):104–112. 10.1016/j.yexcr.2017.01.022 28161537

[28] Malueka RG : Integrated Datalog MicroRNA and Glioma Grade.[Dataset]. *Harvard Dataverse.* 2024;V1. 10.7910/DVN/N3WTWV

