# Supplementary material for: Analysis of Circulating Plasma MicroRNA Profile in Low-Grade and High-Grade Glioma – A Cross-Sectional Study
Source: F1000Res. 2024 Nov 13;13:1361. [Version 1] doi: 10.12688/f1000research.153731.1 (PMC11725040; doi:10.12688/f1000research.153731.1)
Supplement: Peer review history

# Reviewer report: Reviewer response for version 1

**DOI:** 10.5256/f1000research.168661.r353045

**Published:** 2025-01-11

**Authors:** Nirenjen Shanmugasundaram, Sandhanam K

**Affiliations:** 1 Pharmacology, SRM Institute of Science and Technology (Deemed to be University) (Ringgold ID: 93104), Kattankulathur, Tamil Nadu, India

The manuscript titled *"*Analysis of Circulating Plasma MicroRNA Profile in Low-Grade and High-Grade Glioma – A Cross-Sectional Study" presents a valuable contribution to glioma research by exploring a non-invasive diagnostic approach using circulating plasma miRNAs. The identification of miR-518b, miR-1271-3p, and miR-598-3p as potential biomarkers offers a promising alternative to invasive diagnostic techniques, addressing a critical need in neuro-oncology. The study’s methodology is well-detailed, and the results are statistically significant, showcasing the potential of these miRNAs in differentiating glioma grades. This research is particularly important given the challenges of early and accurate glioma diagnosis and the limitations of current diagnostic procedures.

 However, there are a few clarifications and improvements that could enhance the study's rigor and reliability.

 The sample size of 18 glioma patients and 6 controls is limited, which affects the robustness of the results. Expanding the cohort or conducting a validation study would strengthen the findings and improve reliability.

 Additionally, there is inconsistency in the use of the 2021 WHO CNS tumor classification, which should be uniformly applied throughout the manuscript for clarity.

 Some references are outdated, and recent studies on miR-518b and its role in gliomas should be incorporated to provide a more comprehensive discussion.

 The statistical analysis raises concerns due to the small sample size, which may increase the risk of Type I and Type II errors.

 The rationale for using parametric and non-parametric tests should be explained more thoroughly, and additional validation techniques, such as bootstrapping, could be considered.

 Moreover, the manuscript lacks key clinical variables, such as Overall Survival (OS), Progression-Free Survival (PFS), and tumor location. Including these variables in the patient data and correlating them with miRNA expression would significantly enhance the study's clinical relevance.

 The discussion section does not adequately explain why the selected miRNAs are upregulated in low-grade gliomas compared to high-grade gliomas. Providing mechanistic insights or hypotheses linking these findings to glioma biology would improve the study’s impact. Furthermore, the quality control measures for RNA samples stored for over three years need elaboration to ensure data integrity. The inclusion and exclusion criteria for patient selection should also be clearly outlined. Lastly, the study's limitations, such as the small sample size and single-center nature, should be explicitly acknowledged.

 Addressing these clarifications will significantly improve the manuscript's quality and its potential impact on glioma research and clinical diagnostics.

Is the work clearly and accurately presented and does it cite the current literature?

No

If applicable, is the statistical analysis and its interpretation appropriate?

Yes

Are all the source data underlying the results available to ensure full reproducibility?

Yes

Is the study design appropriate and is the work technically sound?

Yes

Are the conclusions drawn adequately supported by the results?

Yes

Are sufficient details of methods and analysis provided to allow replication by others?

Yes

Reviewer Expertise:

Endocrine disorders, wound healing, Diabetes, Nanofibres

We confirm that we have read this submission and believe that we have an appropriate level of expertise to confirm that it is of an acceptable scientific standard.

# Reviewer report: Reviewer response for version 1

**DOI:** 10.5256/f1000research.168661.r353043

**Published:** 2025-01-10

**Authors:** Karla F Meza-Sosa

**Affiliations:** 1 Instituto Nacional de Neurología y Neurocirugía Manuel Velasco Suárez (INNNMVS), Mexico City, Mexico

In this article, the authors collected plasma samples from healthy controls and 18 people diagnosticated with either low-grade or high-grade glioma and measured the expression of 798 microRNAs. Even though the sample size is small, interesting results were obtained as 3 plasma miRNAs (miR-518b, miR-1271-3p, and miR-598-3p) apparently have the potential to distinguish between low- and high-grade gliomas. However, the performed statistical tests throughout the manuscript are not clearly defined and explained accordingly to the type of analyzed data. Please talk about data distribution for each experiment and then, using that information justify the used statistical tests.

 Also, more discussion regarding possible molecular mechanisms regulated by the found miRNAs and making hypothesis about them is needed.

 The article is very interesting and promising to consolidate plasma microRNAs levels as a valuable diagnostic, prognostic and/or classification tool for gliomas in this specific population (Indonesian). However, as mentioned in the conclusion of the paper, data should be taken carefully due to sample size.

**Major comments:**

1. It is not very clear if Figure 1 data are based on the NanoString nCounter Expression Assay or if these miRNAs´ expression was validated by performing qPCR after. Please specify it and, if qPCR was not performed, it would be necessary to validate the obtained results at least for miR-518b, miR-1271-3p, and miR-598-3p.

**Minor comments:**

1. Please check and correct grammar and spelling throughout all your manuscript.
2. Please write microRNAs instead of microRNA when corresponding throughout all your manuscript and change verbs to plural conjugation accordingly.

Is the work clearly and accurately presented and does it cite the current literature?

Partly

If applicable, is the statistical analysis and its interpretation appropriate?

Partly

Are all the source data underlying the results available to ensure full reproducibility?

Partly

Is the study design appropriate and is the work technically sound?

Partly

Are the conclusions drawn adequately supported by the results?

Partly

Are sufficient details of methods and analysis provided to allow replication by others?

No

Reviewer Expertise:

non-coding RNAs, microRNAs, long non-coding RNAs, lncRNAs, post-transcriptional regulation of gene expression, cancer

I confirm that I have read this submission and believe that I have an appropriate level of expertise to confirm that it is of an acceptable scientific standard, however I have significant reservations, as outlined above.

# Reviewer report: Reviewer response for version 1

**DOI:** 10.5256/f1000research.168661.r353046

**Published:** 2025-01-08

**Authors:** Hope Richard

**Affiliations:** 1 Virginia Commonwealth University, Richmond, Virginia, USA

The article describes a non-invasive protocol for the diagnosis of glioma through circulating miRNAs. A small group of patients were enrolled to establish if the grade of glioma could be inferred by the circulating miRNA pattern with the conclusion that miR-518b, miR-1271-3p, and miR-598-3p showed some differential expression when comparing low grade and high-grade gliomas. The weaknesses of the study center predominately around the size of the study population. It would also be useful to re-classify the lesions histologically/molecularly using the 5th edition tumor classification criteria.

Is the work clearly and accurately presented and does it cite the current literature?

Yes

If applicable, is the statistical analysis and its interpretation appropriate?

I cannot comment. A qualified statistician is required.

Are all the source data underlying the results available to ensure full reproducibility?

Yes

Is the study design appropriate and is the work technically sound?

Yes

Are the conclusions drawn adequately supported by the results?

Yes

Are sufficient details of methods and analysis provided to allow replication by others?

Yes

Reviewer Expertise:

surgical neuropathology, cancer genetics

I confirm that I have read this submission and believe that I have an appropriate level of expertise to confirm that it is of an acceptable scientific standard.

# Reviewer report: Reviewer response for version 1

**DOI:** 10.5256/f1000research.168661.r348009

**Published:** 2024-12-31

**Authors:** Emiliya Nikolova

**Affiliations:** 1 Department of Medical Chemistry and Biochemistry, Medical University - Sofia, Sofia, Bulgaria; 2 Independent Medico-Diagnostic Laboratory Genome Center Bulgaria, Sofia, Bulgaria

1. Is the work clearly and accurately presented and does it cite the current literature?

 I recommend updating the introduction with the latest research - some references cited are outdated. The authors use the 2021 WHO grading in the introduction but refer to the previous WHO classification in later sections. It is recommended to consistently use the latest 2021 WHO CNS classification throughout the paper. 

 2. Is the study design appropriate and is the work technically sound?

 I would like to ask for additional information regarding the clarification of the ethical approval before sample collection and sample quality control (the authors have used blood samples for the RNA analysis that have been stored for more than three years).

 3 . Are sufficient details of methods and analysis provided to allow replication by others?

 What is the data distribution and how do the authors determine it? Authors used combination of parametric and non-parametric tests in a small sample size, so conclusions drawn from it should be made with caution.

 4. If applicable, is the statistical analysis and its interpretation appropriate?

 I also have some few statistical concerns regarding the data analysis: the 3 groups (6 LGG, 12 HGG and 6 controls) are too small as a sample size and the used statistical tests require a larger cohort to ensure the reliability and validity of results. The risk of Type I and Type II errors are high

 5. Are the conclusions drawn adequately supported by the results?

 The study may not be correctly analyzed statistically.

 I recommend to increase the sample size or to perform an independent validation study of the selected miRs

 Additional questions and recommendations:

 - You should provide inclusion and exclusion criteria

 - Are there any data for the OS and PFS of patients with HGG? Characteristics such as tumour location, IDH, OS, PFS should be also include in the patients' table.

 - Authors stated that “ However, there is no study linking this miRNA (miR-518b) with glioma patients.16” but indeed there are several studies on this. The discussion should be widening including more studies on using miRs for differentiation HGG and LGG as well as discussing the role of the selected miR in brain malignancies.

 - What is the explanation of higher expression of the selected miRs in the LGG compared to controls and HGG?

Is the work clearly and accurately presented and does it cite the current literature?

Partly

If applicable, is the statistical analysis and its interpretation appropriate?

No

Are all the source data underlying the results available to ensure full reproducibility?

Partly

Is the study design appropriate and is the work technically sound?

No

Are the conclusions drawn adequately supported by the results?

No

Are sufficient details of methods and analysis provided to allow replication by others?

No

Reviewer Expertise:

Neuro-oncology, miRNA analysis, biomarker analysis

I confirm that I have read this submission and believe that I have an appropriate level of expertise to state that I do not consider it to be of an acceptable scientific standard, for reasons outlined above.
